# Caution regarding interpretations of intrauterine γ/δ T cells in protection against experimental vaginal candidiasis

**DOI:** 10.1038/s41385-021-00395-6

**Published:** 2021-03-17

**Authors:** Paul L. Fidel

**Affiliations:** Department of Oral and Craniofacial Biology, Louisiana State University Health School of Dentistry, New Orleans, Louisiana USA

I have read with interest the recent publication by Monin et al. “γ/δ T cells compose a developmentally regulated intrauterine population and protect against vaginal candidiasis” in a recent issue of Mucosal Immunology.^1^ While the majority of the results in the manuscript describe an elegant study characterizing developmentally regulated intrauterine γ/δ T cells, the final portion of the manuscript regarding the potential role of intrauterine γ/δ T cells in vulvovaginal candidiasis (VVC) is somewhat concerning. As a long-standing investigator who has studied the pathogenesis of vulvovaginal candidiasis (VVC) for over 30 years through clinical studies and animal models, I feel compelled to comment on the results and interpretations regarding the vaginal candidiasis experiments. Based on data using γ/δ T cell deficient mice (*Tcrd*^*−/*^^−^), the authors conclude that intrauterine γ/δ T cells provide some level of protection against experimental VVC via reduced infiltration of IL-17-dependent neutrophils (Fig. 7).^1^ While the presented results are supportive of the authors’ conclusions, there are several technical concerns and conceptual inaccuracies/omissions that challenge the interpretations made by the authors.

First, *Candida* vaginitis is strictly an infection of the lower genital tract in otherwise healthy immunocompetent women. This is true for humans and for the experimental mouse model. VVC is characterized as a superficial mycological infection with limited fungal invasion into the vaginal epithelium and no migration to the cervix or the uterus. Inflammatory cell infiltrates are subsequently recruited to the vaginal lumen in response to the fungus and are overwhelmingly composed of neutrophils.^2–4^ Hence, there is a lack of rationale to support the hypothesis that intrauterine T cells play a role in host defense against VVC unless they migrate to the vagina. Recognizing that *Tcrd*^−/^^−^ mice will not only lack intrauterine γ/δ T cells but all γ/δ T cells, caution should be taken with the interpretation that intrauterine γ/δ T cells play a role in protection against VVC as inferred by the title of the publication. We would like to point out that similar studies were performed in our laboratory in 2001 with opposite results, which was neither discussed nor cited.^5^ In our studies using *Tcrd*^−/^^−^ mice, we observed reduced vaginal fungal burden thereby concluding that γ/δ T cells exert an immunoregulatory role that limits vaginal host responses to *C. albicans*. A technical distinction with our study is the fact that CFU assays were restricted to vaginal lumen lavage samples, where the organism resides in the experimental mouse model as in women as well. In addition, CFU levels were normalized to a specific volume of vaginal lavage fluid. In the current manuscript the CFUs were assessed using combined uterine and vaginal tissue homogenates that may have diluted or misrepresented the vaginal burden, and were additionally not normalized to any weight or volume of material.

Second, decades of experimental evidence from the mouse model and clinical studies supports the current paradigm that vaginal *Candida* infection results in a pathological inflammatory response mediated by neutrophil recruitment into the vaginal lumen, which is responsible for the characteristic signs and symptoms of VVC. More recent studies demonstrated that the recruited neutrophils are dysfunctional in their ability to kill *C. albicans* due primarily to the presence of a competitive inhibitor of the interaction between the neutrophil and *Candida*, heparin sulfate,^6^ a phenomenon termed neutrophil anergy.^7^ The immunopathogenesis of VVC was not discussed in the current manuscript but is critically important to the interpretations. The increased CFUs shown in the *Tcrd*^−/^^−^ mice were concomitant with evidence of reduced neutrophil levels in cell suspensions from tissue digests compared to WT infected mice. While the neutrophils indeed migrate through the tissue, their endpoint is the vaginal lumen, where the vast majority of the organism resides. Hence, a more relevant readout to correlate with vaginal fungal burden would be the neutrophils in the lumen from vaginal lavage samples. Even if vaginal lumen neutrophils were shown to be reduced in *Tcrd*^−/^^−^ mice, this result would not be expected to have any influence on fungal burden due to impaired antifungal activity of the neutrophils in the vaginal lumen. Instead, reduced neutrophils would be expected to correlate with reductions in other established hallmarks of VVC immunopathogenesis and inflammation (LDH, S100A8, IL-1β). However, these types of conclusions cannot be drawn because the vaginal inflammatory response was not monitored in these studies. This is compounded further by the choice of *C. albicans* isolate used in the experiments, 529 L. This particular *Candida* isolate has been shown to be relatively inert in the VVC mouse model, and avirulent in general,^8,9^ with little to no triggering of the vaginal neutrophil response and associated inflammatory mediators. Hence, the neutrophils present may have been triggered to migrate in the WT mice independent of *C. albicans* with some other reason for the reduced levels in *Tcrd*^−/^^−^ mice. In reality, data presented in Fig. 7 may reflect this because the neutrophil levels were similar in uninfected vs infected WT mice and likely similar as well for uninfected vs infected *Tcrd*^−/^^−^ mice, neither of which was analyzed statistically.

Third, the authors suggest that reduced responses by IL-17-dependent neutrophils in the *Tcrd*^−/^^−^ mice are responsible for observed increased fungal burden, inferring a protective role for neutrophils. Irrespective of the point noted above regarding functionally anergic neutrophils against *C. albicans* during VVC, the issue of a dependence on IL-17, in any regard, is inaccurate. Our laboratory has shown a lack of any role for IL-17 in the neutrophil response during VVC using genetically deficient mouse strains^10^ and Peters and colleagues have confirmed that the IL-17 that is induced during experimental VVC is dispensable in the immunopathogenic response.^11^ Hence, caution should be taken in the interpretations made in the current manuscript.

While we again recognize that the VVC studies in the manuscript are a small portion of an otherwise elegant study describing the developmentally regulated intrauterine γ/δ T cells, the strong inferences made regarding a putative role of γ/δ T cells in protection against VVC by IL-17-dependent neutrophils is unfortunate. These studies may have benefitted from a formal collaboration with colleagues in the field of *Candida* vaginitis whereby discussions regarding optimal experimental design/methods, choice of fungal strains, and consensus outcome measurements would have enhanced the potential to more adequately address the question(s) being investigated.
